# Targeting nuclear import and export in hematological malignancies

**DOI:** 10.1038/s41375-020-0958-y

**Published:** 2020-07-05

**Authors:** Boaz Nachmias, Aaron D. Schimmer

**Affiliations:** Princess Margaret Cancer Centre, University Health Network, Toronto, ON Canada

**Keywords:** Haematological cancer, Cell signalling, Oncogenes

## Abstract

The transport of proteins across the nuclear membrane is a highly regulated process, essential for the cell function. This transport is actively mediated by members of the karyopherin family, termed importins, or exportins, depending on the direction of transport. These proteins play an active part in tumorigenesis, through aberrant localization of their cargoes, which include oncogenes, tumor-suppressor genes and mediators of key signal transduction pathways. Overexpression of importins and exportins is reported in many malignancies, with implications in cell growth and viability, differentiation, drug resistance, and tumor microenvironment. Given their broad significance across tumors and pathways, much effort is being put to develop specific inhibitors as a novel anticancer therapeutics. Already, selinexor, a specific inhibitor of exportin-1 (XPO1), is approved for clinical use. This review will focus on the role of importins and exportins in hematological malignancies. We will discuss current preclinical and clinical data on importins and exportins, and demonstrate how our growing understanding of their functions has identified new therapeutic targets.

## Introduction

The nuclear membrane forms the barrier between protein transcription in the nucleus and protein translation in the cytoplasm, which is key feature of the eukaryotic cell. This separation allows for controlled transport of proteins in and out of the nucleus, which tightly regulates many cell functions. The transport of proteins across the nuclear membrane is mediated by nuclear transport receptors of the karyopherin family, which includes the importin (IPO) α and IPOβ subfamilies. IPOβ subfamily is further divided to IPOs and exportins depending on the direction of transport, in or out of the nucleus, respectively. Dysregulation of the nuclear import and export machinery is common in cancer, resulting in aberrant localization of various proteins, including tumor-suppressor genes and oncogenes that tumorigenesis [1]. Overexpression of IPOs and exportins is reported in numerous malignancies, and is often correlated with poor prognosis and a more aggressive tumor phenotype

This review will focus on the significance of IPOs and exportins in the pathogenesis of various hematological malignancies. We will highlight how dysregulation of nuclear transport activates key signal transduction pathways with implications in cell proliferation, apoptosis, and drug resistance. We will review the current preclinical studies with specific inhibitors to IPOs and exportins and demonstrate how these findings translate to clinical trials.

## Nuclear transport machinery

The nuclear transport machinery has three primary components: (1) the nuclear pore complex (NPC), (2) the nuclear transport receptors, and (3) small Ras-like GTPase RAN that facilitates nuclear protein import and export by providing a gradient across the nuclear membrane and energy for the transport. The NPC is a basket-like structure composed of multiple copies of up to 50 different proteins termed nucleoporins (Nups). Nups are subdivided into scaffold Nups that form a cylindrical channel about 9–10 nm in diameter and 15 nm long [2], and phenylalanine-glycine Nups that anchor the scaffold Nups to the nuclear membrane and form a selective barrier for nuclear transport [3]. Small (<10 nm, MW < 300) molecules passively traverse this complex, but larger cargo requires active, energy dependent, shuttling mediated by nuclear transport receptors. The majority of nuclear transport receptors are members of the karyopherin family of proteins.

Ten human IPOs are known: IPOβ1, transportin (TNPO) 1 and 2, IPO4, 5, 7 8, 9, 11 and 12. The six human exportins are: Chromosome region maintenance 1 (CRM1)/exportin-1 (XPO1), cellular apoptosis susceptibility (CAS/CSEIL), exportin (XPO) 5, 6 and T, and RanBP17 (Table 1). Certain family members, such as IPO13 facilitate bidirectional transport, while the remaining family members have unidirectional transport functions. All IPOβ family members share a similar tandem Huntingtin, Elongation factor 3, protein phosphatase 2A, and yeast kinase TOR1 (HEAT) repeats. One HEAT repeat comprises two α-helices (of 10–20 residues each) that are linked by a short intra-repeat loop. The tandem HEAT repeat fold is inherently flexible and contribute substantially to Ran-controlled cargo recognition and release. IPOβ proteins bind and shuttle their cargo directly, or use IPOα as an adapter protein to bind cargo. The IPOα family includes seven members in humans and functions mainly as an adapter for the carrier protein. Aside from the IPOβ binding (IBB) domain, IPOα bears an armadillo motif that recognize the nuclear localizing signal (NLS) and C-terminal region that binds to the nuclear export factor of IPOα [4].

**Table 1:** Importins and exportins with examples of their known cargoes.

Protein (gene) name	Selected cargoes
Exportins	
Exportin-1 (*XPO1*)	p53, p21, IkB, BCR-ABL, FOXO3a, TOPO IIa, eIF4E, GR
Cellular apoptosis susceptibility (*CAS/ XPO2*)	Importin α
Exportin for tRNA (*XPOT*)	tRNA
Exportin 4 (XPO4) bidirectional	Import: Sox2, SRY Export: SMAD3, eIF5A
Exportin 5 (*XPO5*)	microRNA
Exportin 6 (*XPO6*)	Actin
Exportin 7 (*XPO7*)	Histone 2A, H3, 14-3-3
Importins	
Importin β1 (*KPNB1*)	p65, β-catenin, JAK1, STAT5, cyclin B1
Transportin 1 (*TNPO1*)	FOXO4, FUS, hnRNAPA1
Transportin 2 (*TNPO2*)	Shared cargoes with TNPO1, HuR
Transportin 3 (*TNPO3*)	SRSF1, CIRBP
Importin 4 (*IPO4*)	Vitamin D receptor
Importin 5 (*IPO5*)	IRF3, RASAL2, HPV E5(16E2)
Importin 7 *(IPO7*)	Ribosomal subunits, SMAD3
Importin 8 *(IPO8*)	eIF4E
Importin 9 (*IPO9*)	NUAK1, nuclear actin
Importin 11 (*IPO11*)	UBE2E3, UBE2E1, PTEN, β-catenin
Importin 13 (*IPO13)-*bidirectional	Import: Ubc9, GR, Pax6 Export; eIF1A

**Fig. 1:**
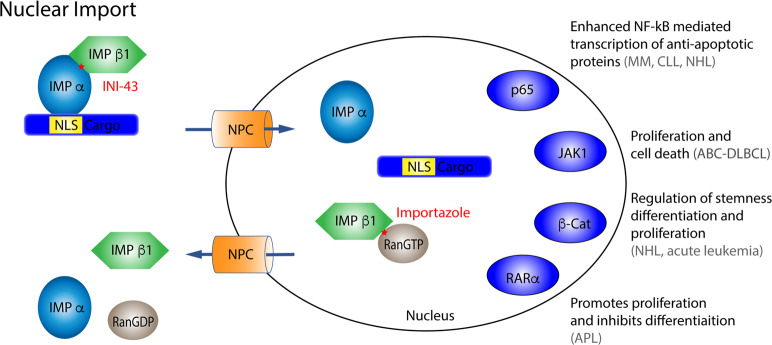
Simplified model of nuclear import with selected hallmark cancer cargo. The importin α/β-cargo complex translocate into the nucleus through the NPC. In the nucleus, binding of abundant RanGTP mediates the release of the cargo. Gray text: hematological malignancies in which the nuclear transport of the specific cargo is shown to be significant. Star marks the binding site of the specified inhibitor in red. NLS nuclear localizing signal, NPC nuclear pore complex, DLBCL diffuse large B-cell lymphoma, APL acute promyelocytic leukemia, MM multiple myeloma, NHL non-Hodgkin lymphoma, CLL chronic lymphocytic leukemia.

The binding of IPOα to NLS-cargo unmasks the IBB domain for IBB to facilitate nuclear import (Fig. 1). Thus, the trimeric complex is needed for successful transport, and eliminates futile transport of empty IPOα/β complex [5]. Next, the IPOβ–IPOα-cargo complex translocates into the nucleus through the NPC. The final key component that allows for the release of the cargo in the nucleus, provide energy for the transport and maintain polarity is the Ras-related small GTPase Ran. In the nucleus abundant GTPase Ran binds to IPOβ, in two regions located between HEAT repeats 1–4 and 12–15, partially overlapping with the IBB domain. Binding of RanGTP acts allosterically on IPOβ inducing conformational changes releasing IPOβ from IPOα. In the cytoplasm, Ran is mainly bound to GDP, thus forming a gradient that directs the import of proteins into the nucleus. The regulator of chromosome condensation 1 (RCC1) reforms RanGTP from RanGDP in the nucleus and plays a critical part in maintaining the gradient across the nuclear membrane [5].

Cargo proteins are recognized by a nuclear localizing sequence or nuclear export sequence (NES). The classical NLS of the SV40-T antigen harbors a short characteristic ~7 basic residues (PKKKRKV) rich in lysine and arginine residues. The NLS of nucleoplasmin is the prototype of the bipartite signal with a small cluster of basic residues positioned 10–12 residues N-terminal to a monopartite-like sequence [6]. Further study revealed more noncanonical NLS, including the 38 residue M9 sequence of hnRNP A1, PTHrP and a proline-tyrosine (PY-NLS) consensus sequence [7]. Each IPO can import a variety of different cargo proteins using different binding sites within the same IPO. A recent study has utilized proximity ligation mass spectrometry based on the BioID system to map the various cargo of each IPO [3]. This allows a better understanding of the specificity and redundancy in nuclear transport by the various IPOs. While a set of distinct cargoes can be identified for each IPO, redundancy exists. Some IPOs such as IPO4 and IPO5 show a significant overlap. Remarkably, functionally related proteins involved in similar biological processes cluster with a specific IPO. In addition, different IPOs bind to different FG-Nups, promoting a highly specific nuclear import. Together the findings suggest a link between function and a designated import cascade [3].

**Fig. 2:**
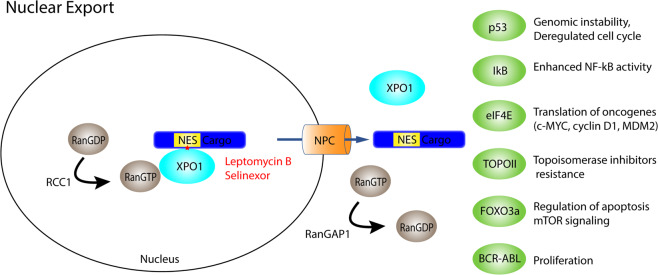
Simplified model of nuclear export with selected cargo of XPO1 XPO1 binds to cargoes bearing NES (nuclear export signal) with RanGTP to facilitate its active transport through the NPC. In the cytoplasm and Ran GTPase-activating protein 1 (RanGAP1) mediates cargo release and RanGTP hydrolysis to RanGDP. The regulator of chromosome condensation 1 (RCC1) reforms RanGTP from RanGDP in the nucleus to maintain the gradient across the nuclear membrane. Star marks the binding site of the specified inhibitor in red. NPC nuclear pore complex.

The classic nuclear export signal consists of a leucin-rich stretch. CRM1/XPO1, the best characterized exportin, has a ring-like shape with a hydrophobic grove formed by HEAT repeats 11 and 12 that recognize and bind leucin-rich NES on cargo proteins [8]. Similar to IPOs, nuclear export by exportin are dependent on RanGTP gradient. However, as opposed to IPOs, exportins recruit their cargo at high RanGTP levels in the nucleus. RanGTP binds XPO1 to facilitate its active transport through the NPC (Fig. 2). In the cytoplasm, RanBP1 and Ran GTPase-activating protein 1 (RanGAP1) mediate cargo release and RanGTP hydrolysis to RanGDP [9].

## Expression and regulation of exportins in hematological malignancies

XPO1 is upregulated in many solid tumors and hematological malignancies and its expression is associated with adverse effect on prognosis [10–12]. For example, myeloma cells have a high XPO1 expression compared to normal plasma cells, and its expression has been shown to increase with progression, to be associated with increased lytic bone lesions and shorter survival [13]. A study that evaluated 511 newly diagnosed AML patients also found high expression of XPO1, that is associated with short survival in multivariate analysis [14]. Similar findings were reported in diffuse large B-cell lymphoma (DLBCL) [15], chronic lymphocytic leukemia (CLL) [16], and T cell lymphoma [17, 18]. In mantle cell lymphoma (MCL), higher XPO1 expression at diagnosis is associated with a poorer prognosis, with a median overall survival of 3.2 years in the low expression *XPO1* cases vs 1.9 years in the high expression XPO1 cases [18].

The regulation of exportin expression is not yet completely understood, and most studies fail to demonstrate a cytogenetic or molecular mutation leading to XPO1 overexpression. On the other hand, hallmark oncogenes such as c-MYC and BCR-ABL directly enhance transcription of XPO1, while p53 negatively regulates XPO1 levels by repressing basal expression and attenuating its induction by c-MYC [19, 20]. Interestingly, the interplay between XPO1 and such oncogenes creates a vicious cycle as XPO1 enhances their activity and in return they support XPO1 expression. Although not common, genetic alterations might also contribute to XPO1 expression. A report in T-ALL discovered a cryptic translocation involving XPO1 and MLL10 with deregulation of HOXA gene locus expression [21]. Copy number gains in the XPO1 locus also occur in primary mediastinal B-cell lymphoma [22].

Finally, several mutations in XPO1 are identified in hematological malignancies. Mutation E571K in XPO1 are found in up 30% of classical Hodgkin disease and primary mediastinal lymphoma. However, the significance of the mutation is still not clear and no correlation with PFS or OS is noted [23, 24]. Missense mutations in XPO1 are reported in a small subset of CLL patients with correlation to unmutated IGHV status, however it is not associated with adverse prognosis [25].

## Pro-tumorigenic pathways involving exportins

As mentioned above, exportins recognize and bind NES-bearing cargoes in the high RanGTP environment of the nucleus. Among XPO1’s cargo are tumor-suppressor proteins (e.g., p53, Rb, p21, p27, APC, and FOXO), mediators of key signal transduction pathways (e.g., IkB), proto-oncogenes (e.g., survivin, BCR-ABL, BRCA1, and Fbw7) and the drug target topoisomerase (Topo) IIα [10, 26].

For example, p53 subcellular localization is tightly regulated in normal cells and governs its function. While it accumulates in the cytoplasm during the G1 phase of cell cycle, p53 enters the nucleus during the G1/S phase transition [27]. Nuclear exclusion of p53 is observed in many tumors and is mediated by XPO1 [28]. Inhibition of XPO1 in AML cells also induces nuclear accumulation of p53, concomitant with decreased growth and viability and induction of differentiation. Accordingly, primary AML cells with defective p53 are much less sensitive to XPO1 inhibition, suggesting the anti-tumorigenic effect of XPO1 is p53 dependent [14]. Similar findings are reported in CLL, multiple myeloma and MCL [13, 17, 18].

High XPO1 expression also supports NF-kB signaling, a key feature in many hematological malignancies, including non-Hodgkin lymphoma, CLL and multiple myeloma. XPO1 mediates the nuclear export of IkB, a key inhibitor of NF-kB transcriptional activity [16, 29, 30]. High expression of XPO1 increases the efflux of IkB, promoting its proteasomal degradation in the cytoplasm, with resulting higher NF-kB activity [31].

Another XPO1 cargo with wide implications in cancer is Topo IIa. Topo IIa nuclear export, mediated by XPO1, does not allow topo II inhibitors such as doxorubicin to induce Topo II/DNA cleavable complexes and resulting apoptosis. XPO1 overexpression thus promotes resistance to Topo inhibitors [32].

Others cargoes of XPO1 are more tumor specific. For example, in AML, the common nucleophosmin 1 (NPM1) mutation promotes the cytoplasmic localization of NPM1 by introducing an XPO1-responsive NES and disrupts the nuclear localization signal [33]. Nuclear re-localization of NPM1 either by genetic manipulation or by inhibiting XPO1 results in loss of HOX genes expression and differentiation of AML cells [34]. AML blasts with cytoplasmic NPM1 are most responsive to XPO1 inhibition [35]. Other examples of tumor- specific cargoes are nuclear export of cyclin D1 mRNA in MCL, with decreased cyclin D1 levels upon inhibition of XPO1 [36, 37], and BCR-ABL in chronic myeloid leukemia (CML), as elaborated below. Finally, nuclear export of signal transducer and activator of transcription (STAT)6 by XPO1 in primary B-cell mediastinal lymphoma augments the Janus Kinase (JAK)/STAT6 signal transduction pathway [38].

In comparison to the wide range of cargoes of XPO1, other exportins show a more restricted range of cargoes. Exportin 2 (XPO2), also called CAS, is a highly interesting protein, as its main cargoes are IPOα [39]. Thus, in essence it regulates nuclear import, and might be target for nuclear import inhibition. High levels of XPO2 are found in many cancer cells lines, including leukemia [40]. However, further studies are needed to clarify its role in cancer biology. The significance of other exportins in cancer is beyond the scope of this review, and readers are referred to the comprehensive review by Cagatay et al. [1].

## Selective inhibitors of nuclear export (SINE)

Given the wide dysregulation of nuclear transport in cancer and their function at the crossroad of many critical signal transduction pathways, targeting exportins has been the focus of many studies in cancer.

Several small molecule inhibitors of XPO1 have been identified. Leptomycin B, initially used as an antifungal agent, was the first XPO1 inhibitor to be reported. Leptomycin B binds to cysteine residue 528 in the hydrostatic grove of XPO1. Further chemical reaction driven by XPO1 and by RanBP1 optimizes the leptomycin B-XPO1 interaction, making it irreversible and potentiates the inactivation of XPO1 by spatial inhibition of NES binding [41, 42]. While numerous studies utilized leptomycin B to inhibit XPO1 in cell lines, allowing the discovery of many of XPO1 cargoes, the molecule is not suitable for clinical use. A single trial with leptomycin B in patients with refractory advanced solid tumors failed to show any efficacy, while marked toxicity of anorexia and malaise were reported [43]. The toxicity observed with leptomycin B is thought to be related to its irreversible binding and inhibition of XPO1, which cripples nuclear export in normal cells as well [44]. As a result of the experiences with Leptomycin B, medicinal chemistry effort focused on developing selective, potent, and reversible inhibitors of XPO1 to produce transient target inhibition and potentially improve the therapeutic window. From these efforts, a series of inhibitors were developed including, KPT-330, also known as selinexor. In preclinical studies, selinexor treatment inhibits XPO1 with nuclear accumulation of its targets. In addition, selinexor reduces XPO1 levels through proteasomal degradation [45]. Studies with Selinexor and other SINEs provide further novel insights on XPO1’s effects in tumor cells. Multiple mechanisms, some interconnected, affecting cell cycle regulation, apoptosis, stress response, and immune evasion are observed. A study in myeloma cells with KPT-185 or KPT-330 shows changes in cell cycle regulators and apoptosis, nuclear accumulation of p53, p27, p21 and FOXO3A, nuclear accumulation of IkB, affecting NF-kB signaling, reduction in c-MYC and antiapoptotic proteins Mcl-1 and Bcl-xL and increase in mRNA of stress response proteins [11, 13]. Interestingly, XPO1 inhibition might also affect the tumor microenvironment with antitumor effects [13].

Consistent with these biological effects, selinexor reduces the growth and viability of many solid tumors, non-Hodgkin lymphoma, myeloma and leukemia cells in vitro and in vivo [16, 30, 46, 47]. Notably, studies in AML and ALL have suggest selinexor also targets the leukemic stem cells, a small subpopulation of leukemia cells, which are thought drive the relapse in AML [48, 49]. Selinexor treatment reduces engraftment in a mouse leukemia xenograft model and reduces frequencies of LSC in selinexor-treated xenografts [50].

Next generation XPO1 inhibitor, KPT-8602, was developed to reduce central nervous system- related toxicity of selinexor, by reducing its blood–brain barrier penetration. KPT-8602 shows similar efficacy to selinexor against AML and ALL cells in vitro and in mouse xenograft models. Initial clinical trials show high antitumor activity and better tolerability [51, 52].

## Preclinical trials combining SINEs with conventional treatments

The understanding of XPO1 regulation of specific cargoes provides the rationale for testing the combination of SINEs with conventional treatments with expected synergism or as a way to overcome resistance.

In vitro studies of XPO1 inhibition in combination with current myeloma treatments find that targeting XPO1 overcomes resistance to proteasome inhibitors. Proteasome inhibitors such as bortezomib and carfilzomib target NF-kB by inhibiting IkB-α proteasomal degradation. Inhibition of XPO1 results in nuclear retention of IkB-α, which might protect it from proteasomal degradation in the cytoplasm. Further studies, performed in myeloma cells from proteasome inhibitor-refractory patients, show that treatment with selinexor results in nuclear retention of IkB-α, along with increase in its total levels. In accord, XPO1 inhibition results in a restored sensitivity to proteasome inhibitors and detection of NF-kB- IkB-α complexes [53]. The combination of selinexor with dexamethasone, which achieved FDA approval, is also based on preclinical studies showing that XPO1 inhibition induces nuclear retention of phosphorylated glucocorticoid receptor, augmenting its activity. Combing dexamethasone with selinexor shows a synergistic effect with inhibition of the mTOR pathway and augmented myeloma cell death [54].

Several preclinical studies have investigated how XPO1 inhibition potentiate common treatment in AML. As mentioned, Topo IIa is one of the cargoes of XPO1, and cytoplasmic localization of Topo II leads to resistance to common Topo inhibitors such as anthracyclines. Treatment with selinexor restores Topo II nuclear localization in AML cell lines and primary AML cells and sensitizes them to treatment with anthracyclines [55]. Another study combines the DNA methyltransferase decitabine with selinexor. While no synergistic effect was noted by combining the two drugs, pretreatment with decitabine sensitizes leukemia cells to selinexor. This effect is presumably by upregulation of key tumor-suppressor genes by decitabine such as CDKN1A and FOXO3A, which are targets of XPO1 [56].

Venetoclax, a selective inhibitor of Bcl-2, is incorporated in the treatment of AML, among other hematological malignancies. Venetoclax treatment reduces the interaction of the proapoptotic protein Bim with BCL-2, however the interaction of Bim with the antiapoptotic protein Mcl-1 is thought to mediate resistance to venetoclax [57]. Treatment with selinexor reduces the levels of Mcl-1, and shows a synergistic effect when combined with venetoclax in AML cells [58].

Targeting XPO1 in AML also shows activity against FLT3-mutated AML cells. FLT3-mutation results in a constitutively active FLT3 signaling, and carries an adverse prognosis with highly proliferative disease. Interestingly, cells with mutated FLT3, regardless of the type of mutation, are more sensitive to XPO1 inhibition, as compared to cells with wild-type FLT3. Further studies into the activity of FLT3 following XPO1 inhibition reveal a surprising compensatory upregulation of phosphorylated FLT3 and its downstream signaling targets. To overcome this, selinexor is combined with FLT3 inhibitor sorafenib, resulting in a marked synergistic effect in vitro and in vivo [59].

In CML, the fusion BCR-ABL protein deregulated kinase activity is mainly limited to the cytoplasm, while treatment with imatinib results in its nuclear localization [60]. Leptomycin B inhibits BCR-ABL export by XPO1 and sensitizes CML cells to imatinib, resulting in reduction in growth and viability [61]. Furthermore, inhibition of XPO1 preferentially targets CD34+ cells form CML patients compared to cord blood control, including CD34+ leukemic blasts from blast crisis CML, which suggests another therapeutic option in this challenging patient population [62].

Treatment of CLL cells with selinexor reduces proliferation of leukemic cells and abrogates phosphorylation of AKT and ERK, reflecting inhibition of BTK activation [63]. Ibrutinib is a BTK inhibitor in clinical use in CLL and other B cell malignancies. Relapse after ibrutinib is often due to the C481S BTK mutation that makes its binding to BTK reversible. Selinexor is also effective in primary CLL cells from patients that relapse after ibrutinib with the C481S mutation [64]. The latter activity of selinexor is also observed in ibrutinib-resistant MCL lines [36].

## Clinical trials with nuclear transport inhibitors

Lessons from preclinical studies with SINEs as single agent and in combinations mentioned above guided the development of clinical studies (Table 2).

**Table 2:** Selected clinical trials with nuclear transport inhibitors in hematological malignancies.

Malignancy	Treatment	Patient characteristics	Number of patients	ORR (%)	Median duration of response	Study, reference
Multiple myeloma	Selinexor	3 or more lines of treatment	57	4		Chen et al. [65]
	Selinexor–dexamethasone	Quad- and penta- refractory	78	21	5 m	Chari et al. [66]
		Triple-class refractory	122	26	4.4 m	Vogl et al. [67]
	Selinexor-bortezoim-dexamethasone	PI sensitive PI refractory	42	84 43	9 m	Bahlis et al. [68]
	Selinexor- carfilzomib-dexamthasone	Median 4 prior treatments	21	48	3 m	Jakubowiak et al. [69]
AML	Selinexor	R/R	95	14	PFS 5.1 vs. 1.3 m OS 9.7 vs. 2.7 m, for responders vs. non-responders	Garzon et al. [72]
	Selinexor-decitabine	Newly dx >60y R/R	25	40		Bhatnagar et al. [73]
	Selinexor-HIDAC-mitoxantrone	Newly dx R/R	12 8	92 38		Wang et al. [74]
	Selinexor-daunorubicin-cytarabine	Newly diagnosed, poor risk	21	53 (42% CR)	Median OS 10.3 m	Sweet et al. [75]
NHL	Selinexor	R/R DLBLC NHL (CLL, MCL, PTCL, FL, BL)	54 25	30	35 m	Kuruvilla et al. [47]

*R*/*R* relapsed refractory, *Dx* diagnosis, *CLL* chronic lymphocytic leukemia, *PTCL* peripheral T cell lymphoma, *MCL* mantle cell lymphoma, *BL* Burkitt’s lymphoma, *PFS* progression free survival, *OS* overall survival, *ORR* overall response rate.

### Multiple myeloma

Selinexor is evaluated in a heavily pretreated MM patients either alone or in combination with dexamethasone. The study includes also a small number of patients with Waldenstrom Macroglobulinemia. Of the 84 patients, overall response rate is 10% with a median duration of response of 5 months. The combination of dexamethasone with selinexor shows a better overall response rate compared with selinexor alone (22% vs 4%) [65]. Selinexor–dexamethasone is further evaluated in triple-class refractory patients shows a partial response in 26% of the patients, with two complete response. Median duration of response is 4.4 months [66]. Similar results are reported from the phase II STORM trial, looking at an even more refractory patient population which progressed on bortezomib, carfilzomib, lenalidomide, and pomalidomide, also reports similar ORR rates (20%), including penta-refractory patients, and similar duration of response (5 months) [67]. The later trial has led to FDA approval of selinexor in combination with dexamethasone for relapsed-refractory multiple myeloma. The selinexor and backbone treatments of multiple myeloma trial is a 9-arm study, evaluating selinexor–dexamethasone in combination with various treatments, including pomalidomide, bortezomib, lenalidomide, ixazomib, and elotuzumab in different combinations (NCT02343042). The results will undoubtfully improve our understanding on how to incorporate selinexor in terms of efficacy and toxicity. The combination of selinexor with low-dose bortezomib (1.3 mg/m^2^) and dexamethasone (SVd) in relapsed refractory MM patients is published, with overall response rate of 63% and 43% for proteasome inhibitor-refractory patients, and PFS of 9 and 6 months, respectively [68]. A different study evaluates selinexor–carfilzomib and dexamethasone, with ORR of 48% in heavily pretreated patients. Interestingly, a very good partial response of 15% is reported for patients who are refractory to carfilzomib in the last line of treatment [69]. Selinexor is also being studied in combination with melphalan before stem cell transplant (NCT02780609).

Common side effects, observed in all clinical trials include hematological toxicity, constitutional and gastrointestinal adverse effects and hyponatremia. Thrombocytopenia is the most common cytopenia, it is dose dependent and occurs in up to 60% of the patients, with 15% grade 3/4 [70]. Interestingly, the mechanism of thrombocytopenia involves the inhibition of megakaryocyte maturation, which might again reflects its ability to target progenitor cells [71]. Constitutional symptoms include nausea, fatigue, anorexia and weight loss and are reported in 40–70% of the patients with most being grades 1–2, as in the STORM study [67]. Grade 3 hyponatremia is reported in 22% of the patients, but is generally asymptomatic and reversible with supportive care. Taken together, dose interruptions for adverse effects are reported in 52% in the STORM study [67]

### Acute leukemia

A phase 1 trial in 95 patients with relapsed refractory AML reports over 50% reduction in bone marrow blasts in 30% of the evaluated patients. The response also translates to an improved PFS (5.1 vs 1.3 months) and OS improvement (9.7 vs. 2.7 months). Common toxicities include fatigue and gastrointestinal, which are primarily grade 1 and 2. The most frequent grades 3 and 4 adverse events are hematological and fatigue. No deaths are reported directly from treatment [72]. Preclinical data highlight several mechanisms of synergism with conventional drugs in AML as well as the ability to overcome resistance. The combination of selinexor with decitabine in relapsed refractory AML patients has an overall response rate of 40%. Adverse events include again hematological toxicities, however a high rate of hyponatremia has been reported [73]. Two trial report the combination of selinexor with high-dose chemotherapy. The combination of high-dose cytarabine and mitoxantrone in 12 newly diagnosed patients and 8 relapsed patients has an ORR rate of 92% and 38%, respectively. Response rate is higher for 80 mg of selinexor compared with 60 mg. Again, myelosuppression is the most common adverse event, with longer than expected count recovery time, resulting in more hemorrhagic and infectious events [74]. Combination of selinexor with 7 + 3 protocol in 21 newly diagnosed poor risk AML patients results in an ORR of 53% with 42% CR rate. Toxicity profile is similar [75]. Further phase 3 studies are necessary to accurately determine the role of selinexor in AML treatment scheme, especially in the newly diagnosed setting. The combination of selinexor with venetoclax is currently evaluated in an on-going study in relapsed refractory patients with AML and non-Hodgkin lymphoma (NCT02485535).

### Non-Hodgkin lymphoma

A phase I study included 79 patients, 54 patients with DLBCL and the rest with other NHL types including: follicular lymphoma, CLL, MCL, cutaneous T cell lymphoma and Burkitt’s lymphoma. Most patients have two or more prior treatments. ORR is ~30% and 4 patients with DLBCL achieve CR, out of 41 evaluated patients. Response rates are similar in germinal center B-cell like (GCB) and non-GCB subtypes. Of six patients with double hit or triple hit lymphoma, 3 respond, 1 with CR. For the patients that achieve CR, response appears durable, with up to 35 months at the time of the report [47]. Selinexor has excellent CNS penetration, and has been evaluated in patients with glioblastoma. A recent case report describes a case of DLBCL with isolated relapse in the CNS. The patient received several lines of treatment including autologous stem cell transplantation prior to the relapse, and failed to respond to methotrexate. Treatment with selinexor results in complete remission within 5 months of treatment [76].

## Key pathways regulated by nuclear import and their significance in hematological malignancies

Overexpression of both IPOα and IPOβ has been reported in non-Hodgkin lymphoma [77], myeloma [78], and leukemia [79] as well as many solid tumors, and is frequently correlated with poor prognosis and a more aggressive tumor phenotype [1, 80]. Further studies have highlighted how nuclear transport affect the function of key oncogenes and tumor-suppressor genes, providing a better understanding of tumor biology and novel targets for therapeutic designs.

One of the best studied examples of how IPOs promote tumorigenesis is the NF-kB pathway. NF-kB are a family of transcription factors whose transcriptional targets promote tumor cells proliferation, inhibit apoptosis and stimulate angiogenesis and tumor invasiveness. Not surprisingly, a high NF-kB activity is observed in solid tumors [81] and hematological malignancies including multiple myeloma, DLBCL, AML, CML, and MDS [82]. The p50/p65 heterodimer is considered the canonical NF-kB transcription factor and its nuclear localization is mediated by IPOβ1. In myeloma cells, genetically silencing IPOβ1 or its inhibition with the IPOβ1 inhibitor importazole (IPZ) reduces the nuclear localization of p65. In accord, the expression of NF-kB signaling target genes are reduced with resulting reduction in growth and viability [78]. IPOβ1 expression in DLBCL is also significantly higher compared with normal B cells, and is associated with higher proliferation, NF-kB activation and shorter overall survival [83].

The Janus Kinase (JAK)/signal transducer and activator of transcription (STAT) pathway is often activated in both myeloid and lymphoid malignancies. Signal transduction is dependent on nuclear import of several of its key players and is dependent on IPOα [84]. JAK1 is especially interesting due its significance in ABC (activated B cell like) DLBCL cells. Silencing of JAK1 reduces viability of DLBCL cells and this effect cannot be rescued by a NLS-mutated JAK1, demonstrating the significance of its nuclear localization [85]. ABC DLBCL has a high relapse rate with adverse prognosis. Thus, dual targeting of the JAK/STAT pathway at the kinase level and by blocking its nuclear import might prove a highly effective therapeutic option. Another example linking nuclear import with key cancer pathways is the wnt pathway. Wnt signal transduction culminates in the IPOβ1-mediated nuclear import of β-catenin [86]. Deregulated wnt signaling has been reported in various lymphoma subtypes, myeloma, and leukemia [87], and depletion of IPOβ1 resulted in G1 arrest and concomitant decrease in β-catenin expression [88]. Finally, IPO7 is linked to B cell receptor (BCR) activation through nuclear shuttling of SYK, which might provide another way to target BCR activation and SYK in hematological malignancies [89].

In leukemia, several studies have demonstrated the significance of nuclear import in driving leukemic pathways. PML/RARa, the hallmark fusion protein of acute promyelocytic leukemia, undergoes cleavage to generate an NLS bearing truncated RARα (NLS-RARα). NLS-RARα is recognized and transported to the nucleus, by the IPOα/IPOβ complex [90], where it promotes proliferation and inhibit differentiation of leukemia cells [91]. Similarly, in *t*(8;21- *AML-1/ETO)* AML, ETO bears a NLS sequence that is recognized by IPOα/β complex and mediates ETO and AML-1/ETO import into the nucleus [92]. High levels of IPOβ1 and its adapter IPOα1 are also detected in bone marrow mononuclear cells derived from CML patients. Silencing of IPOβ1 reduces proliferation and induces apoptosis in CML cells, possibly by decreasing the nuclear localization of the transcription factor E2F1 [93].

Other than the IPOα/β complex, certain IPOs are highlighted in acute leukemia due to their specific cargoes or expression pattern. IPO8 mediates the nuclear localization of Eukaryotic translation initiation factor (eIF4E). eIF4E mediates the nuclear export and cytoplasmic translation of certain mRNAs, many of which are known oncogenes [94]. Silencing of IPO8 results in nuclear accumulation of eIF4E mRNA export targets, such as c-Myc and Mcl-1, with a concomitant reduction in their protein levels. Interestingly, IPO8 is an eIF4E mRNA export target, which further supports the increase of IPO8 levels [95].

Finally, a few sporadic reports link IPOs to acute lymphoblastic leukemia (ALL). A nonsense mutation in MED12, which is part of the transcription-regulation mediator complex, is identified in ALL. The mutation disrupts the NLS sequence of MED12, preventing its recognition by IPOα and its nuclear localization and function [96]. In another study, genetic analysis of ALL blasts prior to treatment found that the IPOβ1 gene contributes to the development of secondary brain tumors [97]. The significance of these reports awaits further studies.

## Development of nuclear import inhibitors

The wide overexpression and significance of IPOα1 and IPOβ1 in numerous malignancies suggests that cancer cells require a higher volume of IPOα/β-mediated traffic across the nuclear membrane. This might reflect a higher proliferative or metabolic state of the cancer cell, or as mentioned above, the transport of cargoes directly involved in the tumor pathogenesis [1, 80]. Given the importance of the nuclear import pathway and the clinical success of inhibiting nuclear export at the level of XPO1, there has been interest in developing nuclear import inhibitors.

As IPOβ1 is a primary regulator of nuclear import and impacts the import of a wide and diverse range of cargo, initial attention focused on developing IPOβ1 inhibitor. Early efforts to develop an IPOβ1 inhibitor sought to target the binding of RanGTP to IPOβ1. A screen for compounds that interfere with the interaction between RanGTP and IPOβ1 identified importazloe. Importazole is a 2,4-diaminoquinazoline that shows high specificity against IPOβ1 and good cell permeability [98]. Importazole has in vitro efficacy in solid tumors and several hematological malignancies including CML and MM [78, 93, 99]. INI-43 (3-(1H-benzimidazol-2-yl)-1-(3-dimethylaminopropyl) pyrrolo[5,4-b]quinoxalin-2-amine) was identified by a structure based in silico screen to identify compounds that bind the overlapping Ran- and IPOα-binding region of IPOβ1. INI-43 inhibits IPOβ1 with a resulting reduction in nuclear localization of p65, an IPOβ1 cargo. Preliminary proof of concept experiments shows that treatment of several solid cancer cell lines, results in cell cycle arrest and cell death. Furthermore, INI-43 treatment inhibits tumor growth in cancer xenograft models, providing a clear evidence of the potential inhibiting nuclear import in cancer [100].

While the transition into clinical studies with import inhibitors has currently not been successful, they hold great promise as anticancer therapeutic approach. One crucial lesson from the development of XPO1 inhibitors is that while irreversible inhibition by leptomycin B produced severe toxicity, development of inhibitors that allow physiological export had much less toxicity, but maintain efficacy in tumor cells. Thus, development of inhibitors that partially inhibit or slow nuclear import might still have effect in a cancer cells, while less toxic to normal tissues. Stepping away from broad inhibition of nuclear import, our growing understanding of how specific IPO and cargo support certain tumors, might allow to develop cargo-specific inhibitors which rely on its unique NLS. Generating small inert peptides with the same NLS competing for IPO binding site, might have a highly specific activity in tumor cells with less toxicity. Another approach might target the interaction of IPO with the NPC. The specificity of IPO–NPC interaction with regard to tumor and cargo is a growing area of study, which might provide the ability selectively inhibit certain cargoes with limited collateral toxicity.

## Conclusion remarks

Transport across the nuclear membrane is at the crossroads of numerous and diverse cellular function, including key elements of cancer development. The proteins that mediate this process, the IPOs, are not merely bystanders but active participants and modulate these processes. Their role in tumorigenesis is evident not only by their wide dysregulation in many hematological malignancies, but also in view of their involvement with key signal transduction pathways, oncogenes, and tumor-suppressor genes. Moreover, data suggest that dysregulation of IPOs and exportins often occur at early stages of tumor development. While there are ample of data on exportins, the role of IPOs is gaining significance as more studies prove their significance. This is especially relevant in treatment combination that target cancer proteins both in their direct function and their subcellular localization. This approach generates a synergistic effect and can also overcome resistance to current treatments. To date, inhibitors of nuclear export are approved for clinical use, but generating potent and specific IPO inhibitors has proven more challenging. Nonetheless, targeting this pathway, both in nuclear import and export still bears great promise as an intervention point with novel therapeutic modalities and merits further study.
